# The impact of coffee consumption on osteoarthritis: insights from NHANES and Mendelian randomization analysis

**DOI:** 10.3389/fnut.2024.1434704

**Published:** 2024-12-02

**Authors:** Kai Feng, Peng Li, Haohui Guo, Zhirong Chen

**Affiliations:** ^1^First Clinical Medical College, General Hospital of Ningxia Medical University, Yinchuan, China; ^2^First Clinical Medical College, Ningxia Medical University, Yinchuan, China; ^3^Department of Orthopedic, General Hospital of Ningxia Medical University, Yinchuan, China

**Keywords:** coffee consumption, osteoarthritis, Mendelian randomization, NHANES, cross-sectional study

## Abstract

**Background:**

Osteoarthritis (OA) is a prevalent degenerative joint condition, and emerging evidence suggests that dietary factors, such as coffee consumption, may influence its risk. However, the relationship between coffee consumption and the risk of developing OA remains ambiguous. This study aims to explore the association between coffee intake and OA complemented by Mendelian randomization (MR) to infer causality.

**Materials and methods:**

We analyzed data from 32,439 participants across 10 NHANES cycles (1999–2018), including 3,676 individuals diagnosed with OA. Osteoarthritis was diagnosed through a structured questionnaire, while coffee consumption was assessed via 24-h dietary recalls. Participants were categorized based on reported coffee intake: 0 cups, <2 cups, 2–4 cups, and >4 cups per day. We employed weighted multivariable logistic regression to examine associations between coffee consumption and OA by using data from the NHANES 1999–2018, adjusting for various covariates. Subsequently, a MR analysis was conducted using genetic variants as instrumental variables to infer causal relationships, with multiple methods including inverse-variance weighted (IVW) analysis, MR-Egger regression, and weighted median techniques to assess the robustness, heterogeneity, and potential pleiotropy of our findings.

**Results:**

Our regression models indicated an increased risk of OA with rising coffee consumption, with significant associations noted particularly for those consuming more than 4 cups daily (OR = 1.19, 95% CI: 1.00–1.41, *p* = 0.049). In MR analysis, coffee intake was causally linked to OA types, demonstrating increased risk for knee OA (KOA: OR = 1.60, 95% CI: 1.08–2.35, *p* = 0.018), hip OA (HOA: OR = 1.85, 95% CI: 1.06–3.25, *p* = 0.031), and combined KOA and HOA (KHOA: OR = 1.66, 95% CI: 1.18–2.33, *p* = 0.003). Sensitivity analyses confirmed the stability of results across multiple evaluation methods.

**Conclusion:**

Our findings highlight a significant association between coffee consumption and an increased risk of OA, suggesting that higher intake levels may contribute to OA morbidity. These results warrant further exploration into the underlying biological mechanisms and implications for dietary guidelines in populations at risk for OA.

## 1 Introduction

Osteoarthritis (OA) is a prevalent degenerative joint disease characterized by the progressive deterioration of cartilage and subchondral bone, leading to pain, stiffness, and functional impairment, especially in the middle-aged and elderly population. According to current global epidemiological reports, the total number of individuals suffering from OA worldwide are estimated to be around 344 million (1, 2). The prevalence of OA among individuals aged 40 and above is approximately 22%. Moreover, as the population continues to age, there is an anticipated annual rise in the prevalence of OA (3, 4). The pathogenesis of OA is complex, observational studies indicate that there are links between obesity (5–7), smoking (8), alcohol user (9), physical exercise (10), and diabetes with OA (11). Recent research has shown a potential link between diet, nutrition and OA, with a growing focus on the impact of coffee consumption (12). Studies (12, 13) have demonstrated that a daily intake of more than 95 milligrams of caffeine is significantly correlated with an increased risk of osteoarthritis (OA). This suggests that the amount found in a standard cup of coffee may be sufficient to elevate this risk. Potential biological mechanisms include direct effects of caffeine on joint tissues, such as enhancing inflammation or influencing the metabolism of the extracellular matrix. Furthermore, caffeine may indirectly impact joint health by affecting blood circulation or promoting bone metabolism. However, the specific mechanisms remain inadequately understood, necessitating further research to elucidate how caffeine influences the development of OA. Previous literature on coffee consumption’s relationship with OA remains inconsistent, prompting further investigations into the specific mechanisms and causal pathways involved.

Among numerous dietary components, coffee has garnered attention due to its widespread consumption and potential health implications. Currently, numerous epidemiological research has shown that consuming more coffee can lower the chances of developing certain illnesses like obesity (13), cardiovascular disease (14), and chronic kidney disease (15). Emerging studies have indicated that coffee consumption may influence the risk of developing OA. For instance, observational studies have shown that increased coffee intake correlates with elevated risk of OA, particularly among individuals exceeding moderate consumption levels (16). However, observational research can be confounded by a myriad of lifestyle variables, such as smoking, alcohol intake, which may skew the causative interpretations of coffee’s effects on OA.

Mendelian randomization (MR) studies, which utilize genetic variants as proxies for exposure, offer a robust alternative to traditional observational approaches by minimizing confounding factors (17, 18). Recent Mendelian studies have explored the association between coffee consumption and various health outcomes, including cardiovascular diseases and metabolic syndromes (19, 20). However, studies exploring the genetic link between coffee consumption and OA are limited (21, 22). Thus, the integration of genetic evidence with epidemiological data becomes paramount for elucidating the causal relationship between coffee intake and OA.

The present study employs data from the National Health and Nutrition Examination Survey (NHANES) along with MR analysis to rigorously assess the impact of coffee consumption on OA risk. By utilizing genetic variants associated with coffee intake, we aim to mitigate confounding influences and provide nuanced insights into the relationship. Given our findings that increased coffee consumption correlates with a higher incidence of both knee and hip OA, the present study opens new avenues for understanding how dietary habits might modulate the risk of chronic joint conditions. In addition to addressing the gaps in existing literature, this research innovates on the conventional methods of establishing causality in dietary epidemiology. Through a comprehensive Mendelian framework that accounts for covariates including body mass index (BMI), dietary components, and comorbidities, our study not only highlights the potential health risks associated with coffee consumption but also lays the groundwork for further investigation into potential therapeutic implications. The findings serve to inform public health strategies and dietary recommendations aimed at minimizing the risk of osteoarthritis while considering the multifactorial nature of this joint disease.

## 2 Materials and methods

### 2.1 Study population in NHANES

**Figure 1:**
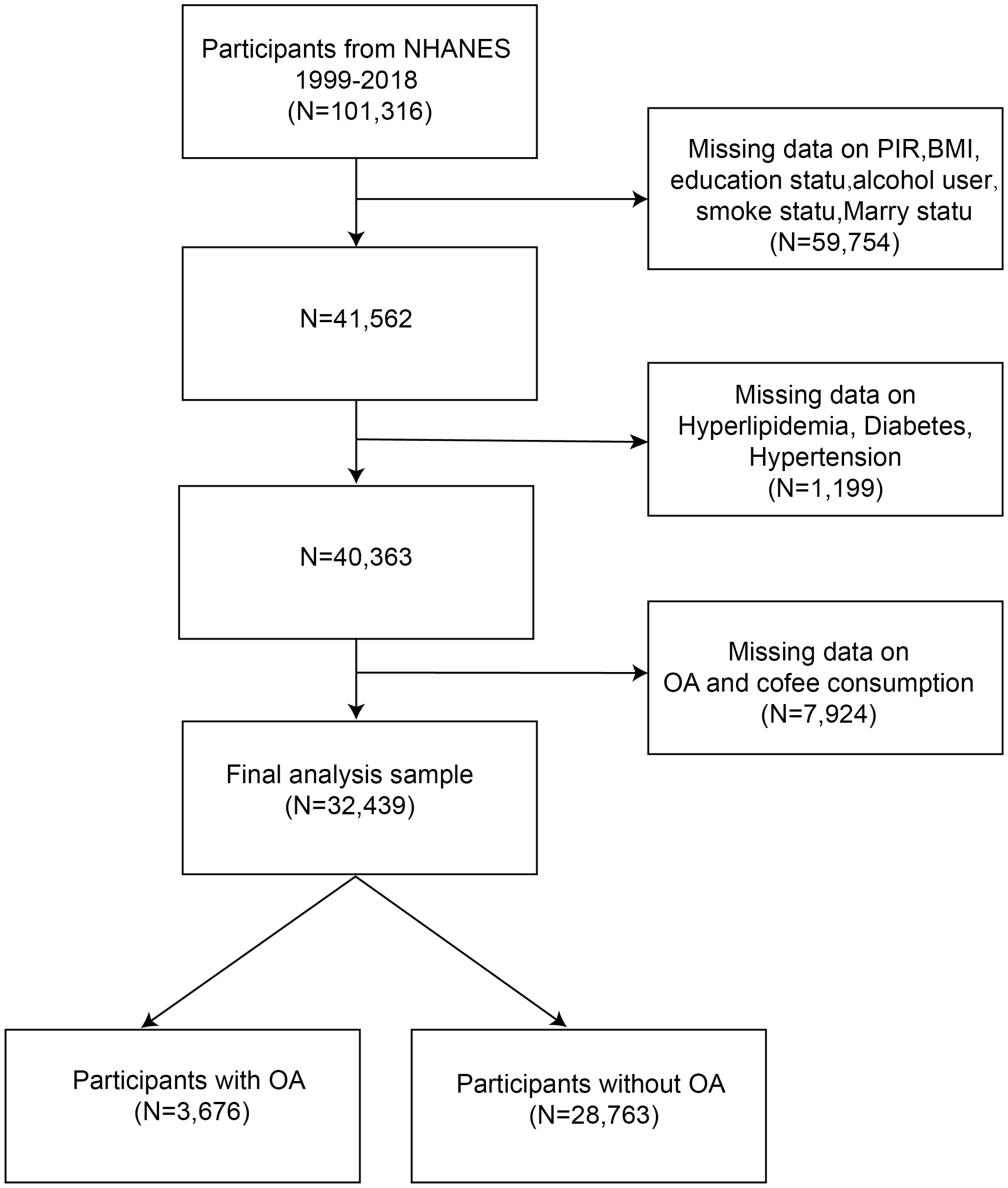
Flowchart of the NHANES study participants.

NHANES is a cross-sectional survey, and the data used in our analyses are publicly available from the NHANES database.^1^ The protocol for the NHANES study was approved by the NCHS Research Ethics Review Board, and all participants provided informed consent. This study analyzed data from 10 rounds of the NHANES survey (1999–2018). Participants were screened based on the following criteria: (1) individuals with missing data on the poverty income ratio (PIR), body mass index (BMI), education status, alcohol use, smoking status, and marital status were excluded (*N* = 59,754); (2) participants missing data on hyperlipidemia, diabetes, or hypertension were excluded (*N* = 1,199); (3) individuals with incomplete data on OA or coffee consumption were excluded (*N* = 7,924). Ultimately, a total of 32,439 participants, including 3,676 individuals diagnosed with OA were included in the analysis (Figure 1).

### 2.2 Diagnosis of OA

OA diagnosis was based on participants’ responses to a medical condition questionnaire administered during NHANES. Participants were asked two questions by a physician: “Has a doctor ever told you that you had arthritis?” and “Which type of arthritis?” Participants were included if they provided a definitive answer to either question and were excluded if they did not (23).

### 2.3 Assessment of coffee consumption

Data on coffee consumption were collected from 24-h dietary recalls and food frequency questionnaires administered within NHANES. The weight of a standard cup of coffee was defined as 283.5 g. Based on reported consumption, participants were categorized into four groups: (1) 0 cups, (2) less than 2 cups, (3) 2 to 4 cups, and (4) more than 4 cups per day (24).

### 2.4 Definition of covariates

The covariates analyzed included age, sex, race, the PIR, marital status, educational attainment, BMI, hypertension, hyperlipidemia, diabetes, alcohol use, and smoking status. Hypertension was defined by one or more of the following criteria: (1) average of three consecutive systolic blood pressures ≥140 mm Hg and/or diastolic blood pressure ≥90 mm Hg; (2) diagnosis by a physician; (3) history of blood pressure-lowering medication use (25). Hyperlipidemia was determined by one or more of the following criteria: (1) triglycerides ≥150 mg/dL; (2) total cholesterol ≥200 mg/dL, LDL-C ≥130 mg/dL, or HDL-C levels <40 mg/dL for men and <50 mg/dL for women; (3) previous use of lipid-lowering drugs (26). Diabetes was defined as one of the following: (1) diagnosis by a physician; (2) history of medication use for blood sugar control (27). Alcohol use was categorized as follows: (1) never: <12 drinks in life; (2) former: at least 12 drinks in a single year but abstinent in the previous year; (3) mild: men ≥2 drinks per day; women ≥1 drink per day; (4) moderate: men ≥3 drinks per day; women ≥2 drinks per day; or drinking ≥2 days to <5 days per month; (5) heavy: men ≥4 drinks per day; women ≥3 drinks per day, or binge drinking on 5 or more days per month (28). Smoking status was classified as (1) never: fewer than 100 cigarettes in lifetime; (2) former: smoked over 100 cigarettes but currently abstaining; (3) current: smoked over 100 cigarettes in lifetime and currently smoking (29).

### 2.5 NHANES analysis

According to the NHANES statistical analysis guidelines, we selected MEC as the test weight for this analysis. We gathered all sample weight information from the Demographic Variables & Sample Weights (DEMO) dataset spanning 1999 to 2018. The sample weights from 1999 to 2002 are designated as wtmec4yr, while those from 2003 to 2018 are wtmec2yr. For this study, the weighted calculation formula used is 2/10 * wtmec4yr + 1/10 * wtmec2yr. Weighted multivariable-adjusted logistic regression was utilized to evaluate the relationship between coffee consumption and OA. Three models were constructed for adjustments: (1) crude model (no adjustment); (2) model 1 (adjusted for age, sex, and race); (3) model 2 (which further adjusted for educational attainment, hypertension, hyperlipidemia, diabetes, alcohol use, and smoking). Statistical analyses were conducted using R version 4.2.2.

### 2.6 Mendelian randomization analysis

The MR analysis utilized genetic variants as instrumental variables (IVs) to assess the causal relationship between coffee intake and OA risk. The analysis followed three core assumptions: (1) the SNPs must be associated with coffee intake; (2) the SNPs must not be affected by confounding factors related to coffee consumption and OA; (3) the SNPs must influence OA only through their relationship with coffee consumption (Supplementary Figure S5). The inverse-variance-weighted (IVW) method was the primary analysis tool, because of its statistical rationality, simplicity of operation, easy interpretation of results, and wide software support, supplemented by MR-Egger regression, weighted median, simple mode, and weighted mode methods, with a focus on identifying potential pleiotropy and detection of horizontal pleiotropy in the selected SNPs (30, 31). The traditional inverse-variance weighted (IVW) method assumes that all instrumental variables affect the outcome solely through the exposure factor, while MR-Egger relaxes this assumption, allowing for the presence of pleiotropy in the instrumental variables. This makes MR-Egger more advantageous when dealing with pleiotropy, as it can provide more accurate estimates of the causal effects (31). Significant results were defined as *p* < 0.05.

### 2.7 Selection of exposure and outcome data

Coffee consumption data derived from a comprehensive analysis of genetic information from participants (*N* = 428,860) in the UK Biobank (Table 1), while summary-level GWAS data on OA was obtained from the same source, encompassing 24,955 cases of knee osteoarthritis (KOA), 15,704 cases of hip osteoarthritis (HOA), and 39,427 cases of knee and hip osteoarthritis (KHOA), alongside 378,169 controls of European descent (32, 33).

**Table 1:** Detailed information on the genome-wide association studies and datasets used in this study.

Trait (s)	Source	GWAS ID	Sample size	SNP
Coffee intake	MRC-IEU	ukb-b-5237	428,860	9,851,867
Knee osteoarthritis	UK Biobank and arcOGEN	ebi-a-GCST007090	403,124	29,999,696
Hip osteoarthritis	UK Biobank and arcOGEN	ebi-a-GCST007091	393,873	29,771,219
Osteoarthritis of the hip or knee	UK Biobank and arcOGEN	ebi-a-GCST007092	417,596	30,265,359
Body mass index	GIANT	ieu-a-2	339,224	2,555,511
Caffeine levels	NA	ebi-a-GCST90026134	291	6,859,792
Mannose	NA	met-a-314	7,793	2,545,645
Omega-3 fatty acid levels	NA	ebi-a-GCST90092931	115,006	11,590,399
Selenium	NA	ieu-a-1075	2,874	2,451,527
Zinc	NA	ieu-a-1079	2,603	2,543,610
Vitamin A	MRC-IEU	ukb-b-9596	460,351	9,851,867
Vitamin C	MRC-IEU	ukb-b-15175	460,351	9,851,867
Vitamin D	MRC-IEU	ukb-b-12648	460,351	9,851,867
Vitamin E	MRC-IEU	ukb-b-12506	460,351	9,851,867

arcOGEN, Arthritis Research UK Osteoarthritis Genetics; SNP, single nucleotide polymorphism.

### 2.8 IV selection

To ensure validity, SNPs for this study were selected with a significance threshold of *p* < 5 × 10^−8^. Pairwise linkage disequilibrium was assessed, with SNPs exhibiting *r*^2^ > 0.001 being filtered out (34). The selected SNPs satisfied the three core assumptions of Mendelian randomization: (1) they are reliably associated with the risk factor of interest (the relevance assumption); (2) they are not associated with any known or unknown confounders (the independence assumption); (3) they influence the outcome only through the risk factor, not via any other causal pathway (the exclusion restriction assumption). *F*-statistic evaluations were performed, excluding those with *F* < 10. The remaining SNPs were analyzed using LDlink^2^ to adjust for confounding variables related to OA, such as obesity, smoking, alcohol use, diabetes, and physical activity.

### 2.9 Statistical analysis

Analytical methods included the IVW approach (35), the weighted median method, MR-Egger regression, simple mode, and weighted mode to explore relationships between coffee intake and OA types. The Cochran *Q* test was employed to investigate heterogeneity among IVs (36). If the *p*-value of the Cochran *Q* test was below 0.05, random-effect models were utilized; otherwise, fixed-effect models were employed. The presence of horizontal pleiotropy was assessed through MR-Egger regression, with a deviation from the origin indicating potential issues with the IVs (37). Using R version 4.2.2 and the TwoSampleMR package, we executed multivariable MR to control for potential confounding variables including BMI, caffeine levels, and various nutrient levels (mannose, omega-3 fatty acids, selenium, zinc, vitamins A, C, D, and E). This approach integrates different phenotypes into the MR analysis as a single exposure.

## 3 Results

### 3.1 Baseline characteristics of participants

This study analyzed data from 10 rounds of the NHANES survey (1999–2018). In our observational study, we included 32,439 individuals with a mean age of 46.55 years (≥20 years). Of these, 16,599 (51.17%) identified as female. The population consisted predominantly of non-Hispanic whites (23,129; 71.3%), with 18,691 (57.62%) being married. Educational attainment showed that 19,992 individuals (61.63%) had completed more than high school. In terms of health metrics, 11,425 participants (35.22%) had a BMI of ≥30. Notably, 17,618 individuals (54.31%) were non-smokers, and 28,890 participants (89.06%) reported alcohol use. Among the cohort, 11,639 individuals were diagnosed with hypertension, 22,402 with hyperlipemia, 2,858 with diabetes, and 3,676 patients with OA. Differences among these variables based on coffee intake were statistically significant (*p* < 0.05) (Table 2).

**Table 2:** Participant characteristics by coffee consumption.

Variable	Total (*N* = 32,439)	Number of cups of coffee				*p*-value
		0 (*N* = 15,630)	0–2 (*N* = 8,136)	2–4 (*N* = 5,552)	>4 (*N* = 3,121)	
Age, mean ± SD	46.55 ± 0.21	42.76 ± 0.23	48.01 ± 0.30	51.04 ± 0.29	51.54 ± 0.34	<0.0001
Sex, *N* (%)						<0.0001
Female	16,599 (51.17)	7,988 (51.11)	4,633 (56.94)	2,847 (51.28)	1,287 (41.25)	
Male	15,840 (48.83)	7,642 (48.89)	3,503 (43.06)	2,705 (48.72)	1,834 (58.75)	
Race, *N* (%)						<0.0001
Mexican American	2,420 (7.46)	1,193 (7.63)	939 (11.54)	316 (5.70)	76 (2.45)	
Non-Hispanic Black	3,299 (10.17)	2,284 (14.61)	818 (10.06)	248 (4.47)	69 (2.22)	
Non-Hispanic White	23,129 (71.30)	10,455 (66.89)	5,022 (61.73)	4,511 (81.25)	2,786 (89.26)	
Other race	3,591 (11.07)	1,698 (10.87)	1,357 (16.67)	477 (8.57)	190 (6.07)	
PIR, *N* (%)						<0.0001
0–1	4,123 (12.71)	2,396 (15.33)	1,112 (13.67)	439 (7.90)	265 (8.50)	
1–3.5	13,790 (42.51)	6,858 (43.88)	3,625 (44.55)	2,177 (39.22)	1,212 (38.83)	
≥3.5	14,526 (44.78)	6,376 (40.79)	3,399 (41.78)	2,936 (52.88)	1,644 (52.67)	
Marital status, *N* (%)						<0.0001
Divorced	3,273 (10.09)	1,371 (8.77)	844 (10.37)	602 (10.84)	420 (13.46)	
Living with partner	2,397 (7.39)	1,197 (7.66)	692 (8.51)	361 (6.51)	180 (5.77)	
Married	18,691 (57.62)	8,321 (53.24)	4,556 (56.00)	3,602 (64.87)	2,058 (65.93)	
Never married	5,680 (17.51)	3,736 (23.90)	1,276 (15.68)	556 (10.02)	246 (7.89)	
Separated	723 (2.23)	352 (2.25)	199 (2.44)	113 (2.03)	67 (2.15)	
Widowed	1,675 (5.16)	653 (4.18)	569 (7.00)	318 (5.73)	150 (4.80)	
Education level, *N* (%)						<0.0001
<High school	3,688 (11.37)	1,877 (12.01)	1,126 (13.84)	456 (8.21)	298 (9.55)	
High school	5,807 (17.90)	3,048 (19.50)	1,303 (16.01)	887 (15.98)	564 (18.06)	
>High school	19,992 (61.63)	9,403 (60.16)	4,850 (59.61)	3,676 (66.21)	1,987 (63.68)	
High school graduate/GED	2,952 (9.10)	1,302 (8.33)	857 (10.54)	533 (9.60)	272 (8.71)	
BMI, *N* (%)						<0.0001
≤25	10,312 (31.79)	5,069 (32.43)	2,623 (32.24)	1,752 (31.55)	903 (28.92)	
25–30	10,702 (32.99)	4,812 (30.79)	2,773 (34.08)	1,927 (34.72)	1,150 (36.86)	
≥30	11,425 (35.22)	5,749 (36.78)	2,740 (33.68)	1,873 (33.73)	1,068 (34.22)	
Hypertension, *N* (%)						<0.0001
No	20,800 (64.12)	10,499 (67.17)	5,011 (61.59)	3,386 (60.99)	1,926 (61.72)	
Yes	11,639 (35.88)	5,131 (32.83)	3,125 (38.41)	2,166 (39.01)	1,195 (38.28)	
Hyperlipidemia, *N* (%)						<0.0001
No	10,037 (30.94)	5,217 (33.38)	2,508 (30.82)	1,556 (28.03)	821 (26.32)	
Yes	22,402 (69.06)	10,413 (66.62)	5,628 (69.18)	3,996 (71.97)	2,300 (73.68)	
Diabetes, *N* (%)						<0.0001
No	29,581 (91.19)	14,403 (92.15)	7,341 (90.23)	5,023 (90.48)	2,819 (90.31)	
Yes	2,858 (8.81)	1,227 (7.85)	795 (9.77)	529 (9.52)	302 (9.69)	
Alcohol use, *N* (%)						<0.0001
Former	4,532 (13.97)	2,246 (14.37)	1,044 (12.83)	683 (12.30)	531 (17.01)	
Heavy	6,786 (20.92)	3,570 (22.84)	1,552 (19.08)	989 (17.81)	673 (21.56)	
Mild	11,889 (36.65)	4,973 (31.82)	3,197 (39.30)	2,430 (43.77)	1,233 (39.51)	
Moderate	5,683 (17.52)	2,501 (16.00)	1,456 (17.89)	1,115 (20.08)	586 (18.77)	
Never	3,549 (10.94)	2,340 (14.97)	887 (10.90)	335 (6.04)	98 (3.15)	
Smoking status, *N* (%)						<0.0001
Former	8,097 (24.96)	2,968 (18.99)	2,077 (25.53)	1,867 (33.63)	1,041 (33.37)	
Never	17,618 (54.31)	9,711 (62.13)	4,639 (57.02)	2,565 (46.20)	1,010 (32.37)	
Now	6,724 (20.73)	2,951 (18.88)	1,420 (17.45)	1,120 (20.17)	1,070 (34.26)	
OA, *N* (%)						<0.0001
No	28,763 (88.67)	14,169 (90.65)	7,107 (87.35)	4,753 (85.60)	2,636 (84.45)	
Yes	3,676 (11.33)	1,461 (9.35)	1,029 (12.65)	799 (14.40)	485 (15.55)	

Mean ± SD for continuous variables: *p*-value calculated by the weighted linear regression model; *N* (%) for categorical variables: *p*-value calculated by the weighted chi-square test. PIR, ratio of income to poverty; BMI, body mass index; OA, osteoarthritis.

### 3.2 Associations between coffee consumption and OA outcomes

Table 3 demonstrates the correlation between various coffee cup sizes and the relative odds of OA. Multiple logistic regression models revealed that in the crude model, the risk of OA was found to increase with coffee consumption at less than 2 cups per day, between 2 and 4 cups per day, and more than 4 cups per day (*p* < 0.05). Comparing the crude model to model 1, which adjusted for covariate, revealed significant differences (*p* < 0.05). After further adjustments for alcohol use and smoking in model 2, coffee consumption greater than 4 cups per day demonstrated a significant association with OA, yielding an odds ratio (OR) of 1.19 (95% CI, 1.00–1.41, *p* = 0.049). The decrease in the size of the effect in model 2 may be attributed to two variables: hypertension and diabetes (Table 4). This indicates an 18.5% higher risk of developing OA associated with higher coffee consumption.

**Table 3:** Multivariable regression analysis of coffee consumption and osteoarthritis (OA).

Regression model	Coffee cups	OR (95% CI)	*p*	*p* for trend
Crude model	0	ref		
	0–2	1.40 (1.24, 1.59)	<0.0001^*^	
	2–4	1.63 (1.46, 1.82)	<0.0001^*^	
	>4	1.78 (1.55, 2.05)	<0.0001^*^	
				<0.0001^*^
Model 1	0	ref		
	0–2	1.02 (0.89, 1.17)	0.747	
	2–4	1.07 (0.95, 1.19)	0.255	
	>4	1.28 (1.10, 1.50)	0.002^*^	
				0.003^*^
Model 2	0	ref		
	0–2	0.98 (0.86, 1.12)	0.770	
	2–4	0.99 (0.88, 1.10)	0.804	
	>4	1.19 (1.00, 1.41)	0.049^*^	
				0.119

Crude model: no adjustments were made. Model 1: adjusted for age, sex, and race. Model 2: adjusted for age, sex, race, education, hypertension, hyperlipidemia, diabetes, alcohol use, and smoking status. OR, odds ratio; CI, confidence interval. ^*^p < 0.05 and ^**^p < 0.01.

**Table 4:** Subgroup analysis of OA risk across different levels of coffee consumption.

Subgroup	0 OR (95% CI)	0–2 OR (95% CI)	*p*	2–4 OR (95% CI)	*p*	>4 OR (95% CI)	*p*	*p* for trend	*p* for interaction
Age									0.015
20–39	ref	0.71 (0.44, 1.13)	0.149	0.83 (0.49, 1.40)	0.479	1.28 (0.72, 2.27)	0.394	0.904	
40–59	ref	1.22 (0.95, 1.57)	0.114	0.95 (0.77, 1.17)	0.594	1.29 (1.00, 1.66)	0.050	0.175	
≥60	ref	0.91 (0.79, 1.06)	0.213	0.99 (0.84, 1.17)	0.920	0.94 (0.75, 1.18)	0.606	0.718	
Sex									0.604
Male	ref	0.99 (0.77, 1.27)	0.935	1.15 (0.93, 1.42)	0.199	1.31 (1.00, 1.72)	0.054	0.046	
Female	ref	1.00 (0.84, 1.19)	0.985	0.91 (0.78, 1.06)	0.242	1.08 (0.88, 1.33)	0.458	0.986	
BMI									0.123
≤25	ref	0.90 (0.72, 1.14)	0.381	0.94 (0.72, 1.22)	0.630	0.80 (0.57, 1.13)	0.207	0.255	
25–30	ref	1.26 (1.00, 1.59)	0.050	1.01 (0.83, 1.24)	0.905	1.36 (1.02, 1.82)	0.038	0.116	
≥30	ref	0.89 (0.73, 1.09)	0.263	1.04 (0.87, 1.24)	0.679	1.28 (1.00, 1.62)	0.046	0.069	
Race									0.494
Non-Hispanic White	ref	1.00 (0.86, 1.18)	0.961	0.98 (0.86, 1.12)	0.778	1.15 (0.96, 1.38)	0.138	0.259	
Mexican American	ref	0.85 (0.57, 1.27)	0.428	0.88 (0.54, 1.42)	0.592	0.80 (0.44, 1.47)	0.470	0.462	
Non-Hispanic Black	ref	0.87 (0.69, 1.08)	0.208	1.09 (0.77, 1.55)	0.619	1.15 (0.71, 1.86)	0.563	0.787	
Other race	ref	1.20 (0.82, 1.75)	0.359	1.22 (0.80, 1.88)	0.355	1.93 (1.02, 3.65)	0.043	0.042	
Hypertension									0.279
No	ref	1.11 (0.90, 1.37)	0.326	0.92 (0.75, 1.13)	0.442	1.06 (0.82, 1.39)	0.645	0.981	
Yes	ref	0.96 (0.81, 1.13)	0.589	1.04 (0.90, 1.20)	0.611	1.23 (1.01, 1.50)	0.041	0.060	
Diabetes									0.613
No	ref	1.02 (0.89, 1.17)	0.793	0.98 (0.86, 1.12)	0.771	1.14 (0.95, 1.37)	0.169	0.321	
Yes	ref	0.91 (0.68, 1.22)	0.526	1.14 (0.84, 1.54)	0.403	1.36 (0.92, 2.02)	0.122	0.098	

BMI, body mass index; OR, odds ratio; CI, confidence interval.

However, when exploring interactions based on sex, BMI, race, hypertension, and diabetes, coffee consumption’s impact on OA prevalence remained consistent across these demographics (*p* for interaction >0.05) (Table 4).

### 3.3 MR analysis

To investigate the causal relationship between coffee consumption and OA risk proposed by our multivariate regression findings, we employed various assessment methods, including IVW, weighted median, MR-Egger, simple mode, and weighted mode analyses.

**Figure 2:**
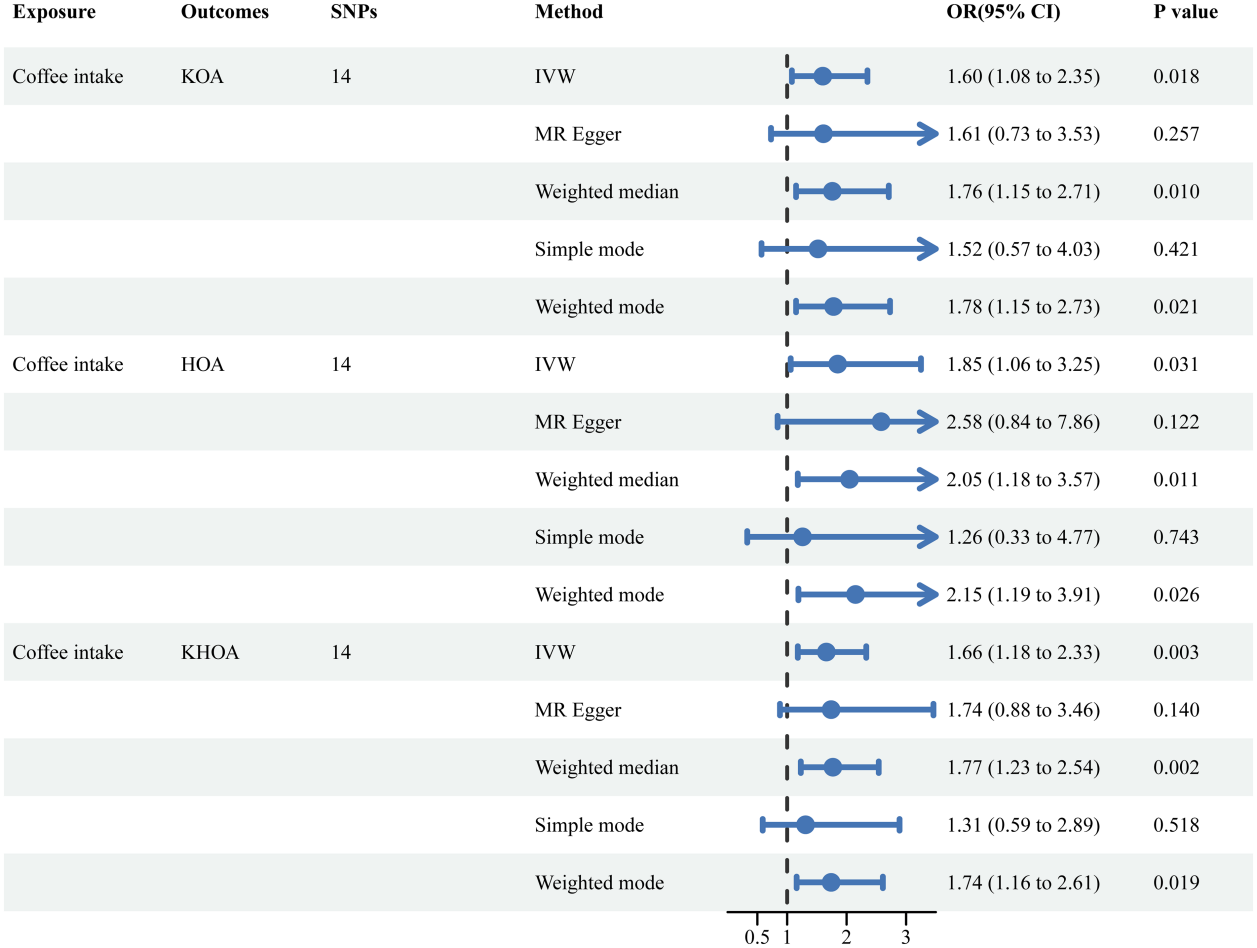
MR analysis investigating the effect of coffee intake on OA.

The IVW analysis indicated that coffee intake is associated with an increased risk of KOA with an OR of 1.60 (95% CI, 1.08–2.35, *p* = 0.018). Similarly, the increased risk for HOA was captured with an OR of 1.85 (95% CI, 1.06–3.25, *p* = 0.031), and for KHOA, the IVW results showed an OR of 1.66 (95% CI, 1.18–2.33, *p* = 0.003) (Figure 2, Supplementary Table S1, and Supplementary Figure S1). Both the weighted median and weighted mode analyses corroborated these findings, with *p*-values below 0.05, reinforcing the suggestion that coffee intake increases morbidity risk (Supplementary Figure S2).

**Figure 3:**
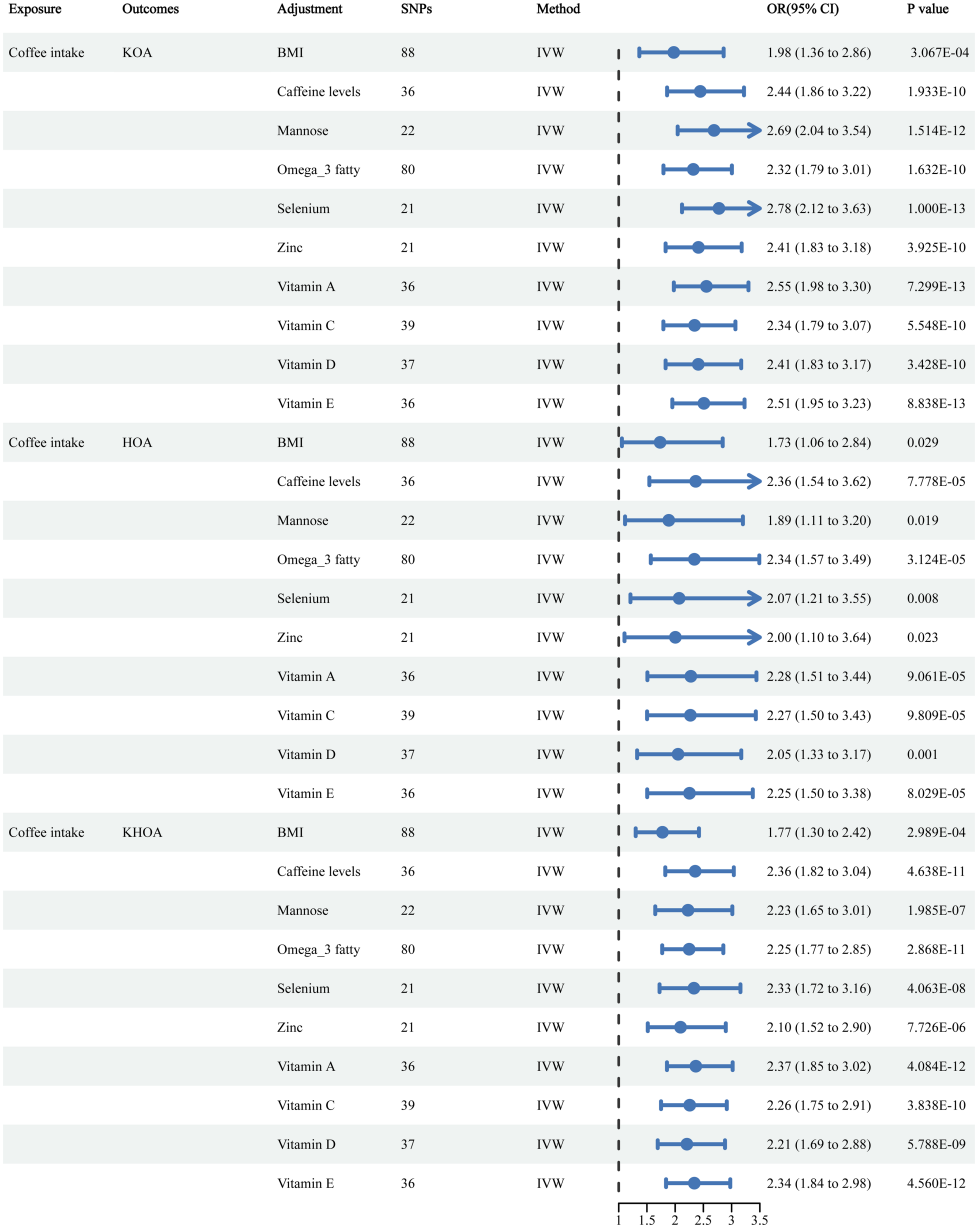
Multivariable MR analysis examining the impact of coffee consumption on the risk of OA.

Funnel plots indicated a lower likelihood of bias affecting these causal associations (Supplementary Figure S3). In sensitivity analyses using the leave-one-out approach, no single SNPs was found to significantly influence causal association estimates (Supplementary Figure S4). The IVW and MR-Egger analyses indicated the absence of heterogeneity or pleiotropy in the association of coffee consumption with KOA, HOA, and KHOA (*p* > 0.05) (Supplementary Table S2), indicating the robustness of our conclusions. In the MVMR analysis, we adjusted for potential confounders including BMI, caffeine levels, mannose, omega-3 fatty acids, selenium, zinc, and vitamins A, C, D, and E. Even after these adjustments, the causal relationships between coffee intake and KOA, HOA, and KHOA remained consistent (Figure 3).

## 4 Discussion

In this study, we utilized observational data from a nationally representative NHANES cohort (1999–2018), supplemented by MR analyses, to investigate the correlation between coffee consumption and the risk of developing OA. Our findings indicated that consumption of coffee exceeding 4 cups per day is associated with an elevated risk of OA. Moreover, the MR analysis corroborated a positive causal relationship, enhancing the validity of observational outcomes.

Exploration of the literature surrounding coffee and OA risk elucidates the complexity of this connection. Coffee as one of the world’s most popular drinks (38), contains a variety of biologically active substances, such as caffeine and polyphenols, which have complex effects on human health (39, 40). While moderate coffee consumption has been linked to improved concentration and digestion, excessive intake may lead to health complications, including heightened anxiety and calcium loss (41–43). Our study uses data from NHANES 1999–2018, multivariate logistic regression analyses found that the risk of developing OA was 78.4% for those with coffee intake greater than 4 cups per day compared to those who did not consume coffee in the crude model, 28.3% for those with coffee intake greater than 4 cups per day compared to those who did not consume coffee in model 1, and 18.5% in model 2. Although we adjusted for age, sex, race, educational attainment, hypertension, hyperlipidemia, diabetes, alcohol user and smoking in model 2 (*p* for trend >0.05), the presence of confounders is common in observational studies (44, 45). Our research also found that the likelihood of developing OA was higher with a daily coffee intake of more than 4 cups (*p* < 0.05). Previous research has explored the relationship between coffee intake and OA, with findings that vary significantly across studies. Mendelian randomization (MR) studies utilize genetic variants, such as single nucleotide polymorphisms (SNPs), as instrumental variables. Variability in the selection of SNPs across different studies may influence the consistency and reliability of their findings. Additionally, sample size plays a crucial role in determining the statistical power of MR studies. Disparities in population characteristics among various studies—along with differences in genetic backgrounds, environmental factors, and lifestyle choices—can further impact the outcomes of MR research. For instance, a Mendelian randomized analysis conducted in 2018 and additional two-sample MR studies reported in 2024 have observed associations between coffee consumption and OA, which appears to have used the different data source and SNPs for GWAS database (21, 22). However, our study distinguishes itself by employing robust regression models and analyzing an updated cohort, thereby refining the understanding of specific types of OA linked to coffee intake.

Caffeine, the principal psychoactive component of coffee, may detrimentally impact joint health through multiple mechanisms, particularly when consumed in excessive amounts. High doses of caffeine are known to interfere with calcium absorption and metabolism, which are critical for maintaining bone density and integrity. This interference can lead to impaired bone health over time (46). Additionally, caffeine may exacerbate joint inflammation by stimulating the production of pro-inflammatory mediators within synovial cells. Increased inflammatory markers can further contribute to the degeneration of articular cartilage, a primary feature of OA (47). Comprehensive reviews, such as those by Guillán-Fresco et al. (48), have noted that caffeine’s negative effects extend to the functions of articular and growth plate cartilages, amplifying susceptibility to OA. Interestingly, the relationship between caffeine dosage and its effects on OA may not be linear. For instance, lower to moderate caffeine intake might have beneficial effects due to the presence of antioxidants, which could mitigate oxidative stress and inflammation. However, higher caffeine consumption has consistently been associated with adverse outcomes, as evidenced by Bang’s et al. (16) findings linking excessive coffee consumption to knee OA among Korean males. Disparities in participant demographics could contribute to these divergent results. The studies referenced involved distinct populations; for example, the research by Bang et al. (16) focused on a Korean demographic, while our analyses employed a diverse U.S. sample. This variation underscores the importance of considering genetic, environmental, and lifestyle factors that may influence the relationship between coffee consumption and OA. Conversely, some studies, such as the one conducted by Wood et al. (49), suggested that coffee consumption may have a preventive effect on OA, particularly in certain demographics. Lim et al. (12) conducted a cross-sectional investigation in a Korean demographic, discovered that women who consumed more coffee had a lower risk of developing OA. These discrepancies can be attributed to variations in study designs, participant characteristics, and methodologies for assessing coffee intake and OA diagnosis (49). The variations observed among these research outcomes could be ascribed to differences in study designs, such as the use of cross-sectional versus longitudinal data—can influence outcomes. To strengthen our conclusions, we employed multiple MR analyses to mitigate residual confounding. Our results reiterated earlier findings from the NHANES analysis, consistently linking coffee intake with increased OA risk, thereby enhancing the reliability of our observations. Moreover, these varying conclusions may arise from differences in methodology, specifically how coffee consumption and OA diagnosis were measured. For instance, participants’ coffee intake was often assessed through self-reported questionnaires, which can lead to recall bias and inaccurate measures, affecting the reliability of findings. Another critical element to consider is the role of confounding variables. While our study adjusted for numerous potential confounders such as age, sex, race, and various health metrics, residual confounding may still exist. Some previous studies, including those conducted by Lim et al. (12), may not have adequately controlled for certain confounding factors (e.g., dietary habits, physical activity, and hormonal levels), leading to inconsistent findings. Our MR approach aimed to mitigate these biases by providing a clearer causal pathway between coffee consumption and OA, yielding consistent results across methodologies.

Our study has several strengths. The large cross-sectional NHANES database, alongside MR analysis, provides a robust platform for examining the pertinent issues of coffee consumption and OA risk. Despite the richness of our findings, the current study is not without limitations. The potential for measurement error in self-reported coffee intake and the reliance on questionnaire-based diagnoses of OA may introduce bias. Moreover, However, there are some limitations. First, cross-sectional data introduce challenges related to the imprecise measurement of coffee intake, possible recall bias, and reliance on self-reported OA diagnoses, which may affect the results. Additionally, while our cross-sectional data is derived from a diverse U.S. population, the MR cohort primarily represents European demographics. This discrepancy highlights the necessity for further research to validate the generalizability of our findings across various ethnicities. Finally, despite controlling for many confounding variables associated with OA, it is crucial to acknowledge that other factors, such as hormonal influences, may still play a role in the observed associations (50). Future research should aim to encompass a broader array of confounders to further elucidate the complex relationship between coffee consumption and OA risk.

## 5 Conclusion

In conclusion, our study significantly contributes to the growing body of literature exploring the relationship between coffee consumption and OA. The findings underscore the importance of considering dietary habits in OA risk assessments, particularly in populations with high coffee consumption. Continued research to explore the underlying biological mechanisms and the implications of coffee intake for OA management is needed to inform dietary recommendations for those at risk of developing this debilitating condition.

## Data Availability

The original contributions presented in the study are included in the article/Supplementary material, further inquiries can be directed to the corresponding author.

## Footnotes

^1^ https://www.cdc.gov/nchs/nhanes/index.htm

^2^ https://ldlink.nci.nih.gov/
