# Supplementary material for: The impact of coffee consumption on osteoarthritis: insights from NHANES and Mendelian randomization analysis
Source: Front Nutr. 2024 Dec 2;11:1434704. doi: 10.3389/fnut.2024.1434704 (PMC11650599; doi:10.3389/fnut.2024.1434704)

Supplementary Material

Supplementary Table S1. Mendelian randomization estimates for associations between coffee intake and OA

Supplementary Table S2. Sensitivity analysis and pleiotropy test between coffee intake and OA

Supplementary Table S3. Detailed information on the instrumental variables for exposures (coffee intake) and outcomes (KOA) in the MR analysis

Supplementary Table S4. Detailed information on the instrumental variables for exposures (coffee intake) and outcomes (HOA) in the MR analysis

Supplementary Table S5. Detailed information on the instrumental variables for exposures (coffee intake) and outcomes (KHOA) in the MR analysis

Supplementary Figure S1. Forest plots of causal associations between exposures and outcomes.

Supplementary Figure S2. Scatter plots of causal associations between exposures and outcomes.

Supplementary Figure S3. Funnel plots of causal associations between exposures and outcomes.

Supplementary Figure S4. Leave-one-out analysis plots of causal associations between exposures and outcomes

Supplementary Figure S5. The basic assumptions of Mendelian randomization (MR).

Table S1. Mendelian randomization estimates for associations between coffee intake and OA

| Outcomes | SNPs | Method | OR | 95% LCI | 95% UCI | P value |
| --- | --- | --- | --- | --- | --- | --- |
| KOA | 14 | IVW | 1.60 | 1.08 | 2.35 | 0.018 |
|  |  | MR Egger | 1.61 | 0.73 | 3.53 | 0.257 |
|  |  | Weighted median | 1.76 | 1.15 | 2.71 | 0.010 |
|  |  | Simple mode | 1.52 | 0.57 | 4.03 | 0.421 |
|  |  | Weighted mode | 1.78 | 1.15 | 2.73 | 0.021 |
| HOA | 14 | IVW | 1.85 | 1.06 | 3.25 | 0.031 |
|  |  | MR Egger | 2.58 | 0.84 | 7.86 | 0.122 |
|  |  | Weighted median | 2.05 | 1.18 | 3.57 | 0.011 |
|  |  | Simple mode | 1.26 | 0.33 | 4.77 | 0.743 |
|  |  | Weighted mode | 2.15 | 1.19 | 3.91 | 0.026 |
| KHOA | 14 | IVW | 1.66 | 1.18 | 2.33 | 0.003 |
|  |  | MR Egger | 1.74 | 0.88 | 3.46 | 0.140 |
|  |  | Weighted median | 1.77 | 1.23 | 2.54 | 0.002 |
|  |  | Simple mode | 1.31 | 0.59 | 2.89 | 0.518 |
|  |  | Weighted mode | 1.74 | 1.16 | 2.61 | 0.019 |

Abbreviations: KOA, Knee osteoarthritis; HOA, Hip osteoarthritis; KHOA, Osteoarthritis of the hip and/or knee; IVW, Inverse variance weighting; OR, Odds Ratio; 95% LCI, Lower 95% confidence interval; 95% UCI, Upper 95% confidence interval; SNPs, Single nucleotide polymorphisms.

Table S2. Sensitivity analysis and pleiotropy test between coffee intake and OA

| Outcome |  | KOA | HOA | KHOA |
| --- | --- | --- | --- | --- |
| IVW  (heterogeneity) | P value | 0.207 | 0.051 | 0.118 |
|  | Q | 16.832 | 22.288 | 19.156 |
| MR Egger  (heterogeneity) | P value | 0.156 | 0.044 | 0.086 |
|  | Q | 16.831 | 21.474 | 19.115 |
| MR Egger  (pleiotropy) | P value | 0.981 | 0.513 | 0.874 |
|  | intercept | -0.0002 | -0.007 | -0.001 |

Abbreviations: KOA, Knee osteoarthritis; HOA, Hip osteoarthritis; KHOA, Osteoarthritis of the hip and/or knee; MR, Mendelian randomization; IVW, Inverse variance weighting.

Table S3. Detailed information on the instrumental variables for exposures (coffee intake) and outcomes (KOA) in the MR analysis

| SNP | EA | OA | eaf.exposure | eaf.outcome | beta.exposure | beta.outcome | se.exposure | se.outcome | PVE | F |
| --- | --- | --- | --- | --- | --- | --- | --- | --- | --- | --- |
| rs1057868 | T | C | 0.284986 | 0.2846 | 0.0199509 | 0.0259 | 0.00178517 | 0.0103 | 0.000291155 | 124.9009737 |
| rs117810762 | A | G | 0.017881 | 0.0178 | 0.0359086 | -0.0408 | 0.00617871 | 0.0356 | 7.88E-05 | 33.77546389 |
| rs117968677 | A | G | 0.024207 | 0.0244 | -0.0310299 | 0.0494 | 0.00551601 | 0.0314 | 7.38E-05 | 31.6454053 |
| rs12989746 | T | G | 0.249928 | 0.25 | 0.01035 | 0.0093 | 0.00186429 | 0.0107 | 7.19E-05 | 30.8214992 |
| rs2597805 | T | C | 0.682463 | 0.6843 | 0.00985502 | -0.0082 | 0.00175623 | 0.0101 | 7.34E-05 | 31.48851914 |
| rs4410790 | C | T | 0.632141 | 0.6325 | 0.039072 | 0.0226 | 0.00167288 | 0.0096 | 0.001270381 | 545.5087317 |
| rs57918684 | A | G | 0.154747 | 0.1551 | 0.0128864 | -0.0014 | 0.00223845 | 0.0129 | 7.73E-05 | 33.14121451 |
| rs6062682 | T | C | 0.464546 | 0.4644 | 0.0103704 | 0.0084 | 0.00163929 | 0.0094 | 9.33E-05 | 40.02021679 |
| rs61928609 | C | A | 0.835328 | 0.835 | -0.0147305 | -7.0E-04 | 0.00217536 | 0.0125 | 0.000106908 | 45.85352204 |
| rs73075167 | T | A | 0.12918 | 0.1303 | -0.0160639 | -0.0038 | 0.00244429 | 0.014 | 0.000100702 | 43.19132446 |
| rs75347775 | A | G | 0.244531 | 0.2442 | 0.0104504 | 0.0249 | 0.00187898 | 0.0108 | 7.21E-05 | 30.93296233 |
| rs7811609 | T | C | 0.374746 | 0.3742 | 0.00913864 | -0.0064 | 0.00166468 | 0.0096 | 7.03E-05 | 30.13711083 |
| rs78267637 | G | C | 0.038115 | 0.0389 | -0.0254259 | -0.0072 | 0.00431659 | 0.0245 | 8.09E-05 | 34.69533367 |
| rs8056750 | T | C | 0.359129 | 0.3591 | 0.0105333 | 0.0054 | 0.00173692 | 0.01 | 8.57E-05 | 36.77640515 |

Table S4. Detailed information on the instrumental variables for exposures (coffee intake) and outcomes (HOA) in the MR analysis

| SNP | EA | OA | eaf.exposure | eaf.outcome | beta.exposure | beta.outcome | se.exposure | se.outcome | PVE | F |
| --- | --- | --- | --- | --- | --- | --- | --- | --- | --- | --- |
| rs1057868 | T | C | 0.284986 | 0.285 | 0.0372 | 0.284986 | 0.00178517 | 0.0129 | 0.000291155 | 124.9009737 |
| rs117810762 | A | G | 0.017881 | 0.0179 | 0.0035 | 0.017881 | 0.00617871 | 0.0444 | 7.88E-05 | 33.77546389 |
| rs117968677 | A | G | 0.024207 | 0.0243 | 0.0902 | 0.024207 | 0.00551601 | 0.0397 | 7.38E-05 | 31.6454053 |
| rs12989746 | T | G | 0.249928 | 0.2498 | -9E-04 | 0.249928 | 0.00186429 | 0.0134 | 7.19E-05 | 30.8214992 |
| rs2597805 | T | C | 0.682463 | 0.6849 | 0.01 | 0.682463 | 0.00175623 | 0.0127 | 7.34E-05 | 31.48851914 |
| rs4410790 | C | T | 0.632141 | 0.6321 | 0.0318 | 0.632141 | 0.00167288 | 0.0121 | 0.001270381 | 545.5087317 |
| rs57918684 | A | G | 0.154747 | 0.1551 | -0.0168 | 0.154747 | 0.00223845 | 0.0161 | 7.73E-05 | 33.14121451 |
| rs6062682 | T | C | 0.464546 | 0.4639 | 0.0025 | 0.464546 | 0.00163929 | 0.0118 | 9.33E-05 | 40.02021679 |
| rs61928609 | C | A | 0.835328 | 0.8348 | -0.0012 | 0.835328 | 0.00217536 | 0.0157 | 0.000106908 | 45.85352204 |
| rs73075167 | T | A | 0.12918 | 0.13 | -0.0416 | 0.12918 | 0.00244429 | 0.0176 | 0.000100702 | 43.19132446 |
| rs75347775 | A | G | 0.244531 | 0.2435 | -0.0171 | 0.244531 | 0.00187898 | 0.0136 | 7.21E-05 | 30.93296233 |
| rs7811609 | T | C | 0.374746 | 0.3746 | 0.0108 | 0.374746 | 0.00166468 | 0.012 | 7.03E-05 | 30.13711083 |
| rs78267637 | G | C | 0.038115 | 0.0389 | -0.0043 | 0.038115 | 0.00431659 | 0.0308 | 8.09E-05 | 34.69533367 |
| rs8056750 | T | C | 0.359129 | 0.3583 | -0.0042 | 0.359129 | 0.00173692 | 0.0126 | 8.57E-05 | 36.77640515 |

Table S5. Detailed information on the instrumental variables for exposures (coffee intake) and outcomes (KHOA) in the MR analysis

| SNP | EA | OA | eaf.exposure | eaf.outcome | beta.exposure | beta.outcome | se.exposure | se.outcome | PVE | F |
| --- | --- | --- | --- | --- | --- | --- | --- | --- | --- | --- |
| rs1057868 | T | C | 0.284986 | 0.285 | 0.0199509 | 0.0295 | 0.00178517 | 0.0084 | 0.000291155 | 124.9009737 |
| rs117810762 | A | G | 0.017881 | 0.0179 | 0.0359086 | -0.0141 | 0.00617871 | 0.029 | 7.88E-05 | 33.77546389 |
| rs117968677 | A | G | 0.024207 | 0.0244 | -0.0310299 | 0.0596 | 0.00551601 | 0.0257 | 7.38E-05 | 31.6454053 |
| rs12989746 | T | G | 0.249928 | 0.2499 | 0.01035 | 0.0051 | 0.00186429 | 0.0088 | 7.19E-05 | 30.8214992 |
| rs2597805 | T | C | 0.682463 | 0.6844 | 0.00985502 | 3.00E-04 | 0.00175623 | 0.0083 | 7.34E-05 | 31.48851914 |
| rs4410790 | C | T | 0.632141 | 0.6326 | 0.039072 | 0.0227 | 0.00167288 | 0.0079 | 0.001270381 | 545.5087317 |
| rs57918684 | A | G | 0.154747 | 0.155 | 0.0128864 | -0.0067 | 0.00223845 | 0.0105 | 7.73E-05 | 33.14121451 |
| rs6062682 | T | C | 0.464546 | 0.4643 | 0.0103704 | 0.0074 | 0.00163929 | 0.0077 | 9.33E-05 | 40.02021679 |
| rs61928609 | C | A | 0.835328 | 0.835 | -0.0147305 | -0.0019 | 0.00217536 | 0.0102 | 0.000106908 | 45.85352204 |
| rs73075167 | T | A | 0.12918 | 0.13 | -0.0160639 | -0.0188 | 0.00244429 | 0.0115 | 0.000100702 | 43.19132446 |
| rs75347775 | A | G | 0.244531 | 0.2441 | 0.0104504 | 0.0088 | 0.00187898 | 0.0088 | 7.21E-05 | 30.93296233 |
| rs7811609 | T | C | 0.374746 | 0.3743 | 0.00913864 | 3.00E-04 | 0.00166468 | 0.0078 | 7.03E-05 | 30.13711083 |
| rs78267637 | G | C | 0.038115 | 0.0388 | -0.0254259 | -0.0053 | 0.00431659 | 0.0201 | 8.09E-05 | 34.69533367 |
| rs8056750 | T | C | 0.359129 | 0.3589 | 0.0105333 | 0.0026 | 0.00173692 | 0.0082 | 8.57E-05 | 36.77640515 |

Figure S1. Forest plots of causal associations between exposures and outcomes. (A) Forest plot between coffee intake and KOA; (B) Forest plot between coffee intake and HOA; (C) Forest plot between coffee intake and KHOA.


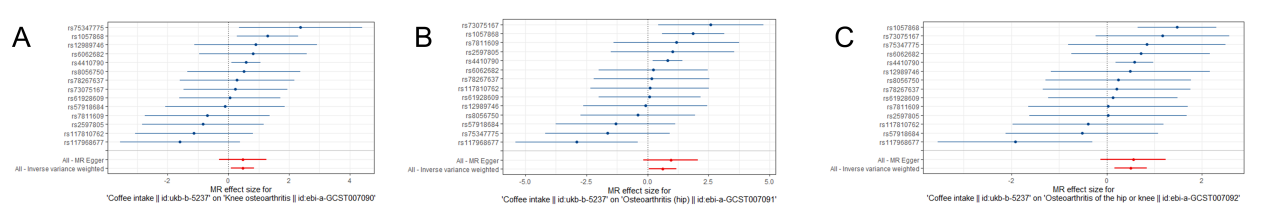


Figure S2. Scatter plots of causal associations between exposures and outcomes. (A) Scatter plot between coffee intake and KOA; (B) Scatter plot between coffee intake and HOA; (C) Scatter plot between coffee intake and KHOA.


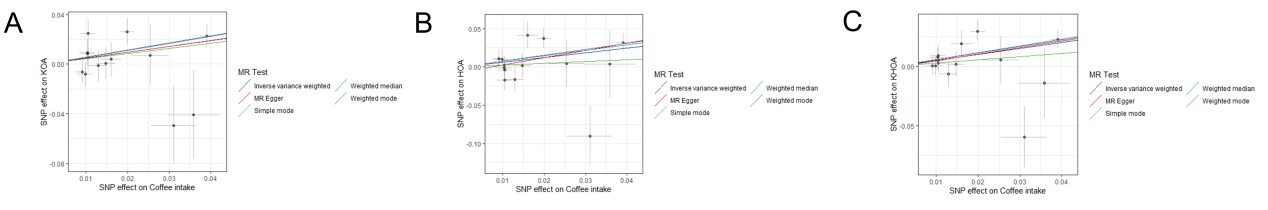


Figure S3. Funnel plots of causal associations between exposures and outcomes. (A) Funnel plot between coffee intake and KOA; (B) Funnel plot between coffee intake and HOA; (C) Funnel plot between coffee intake and KHOA.


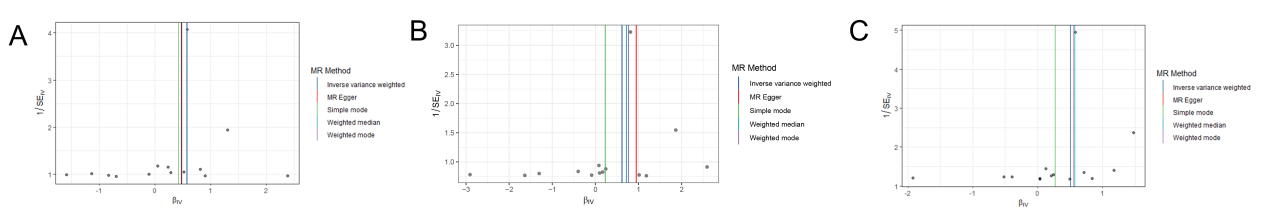


Figure S4. Leave-one-out analysis plots of causal associations between exposures and outcomes. (A) Leave-one-out analysis plot between coffee intake and KOA; (B) Leave-one-out analysis plot between coffee intake and HOA; (C) Leave-one-out analysis plot between coffee intake and KHOA.


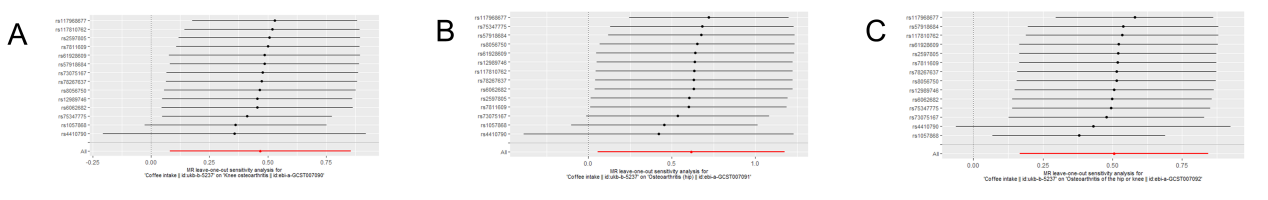


Figure S5. The basic assumptions of Mendelian randomization (MR).


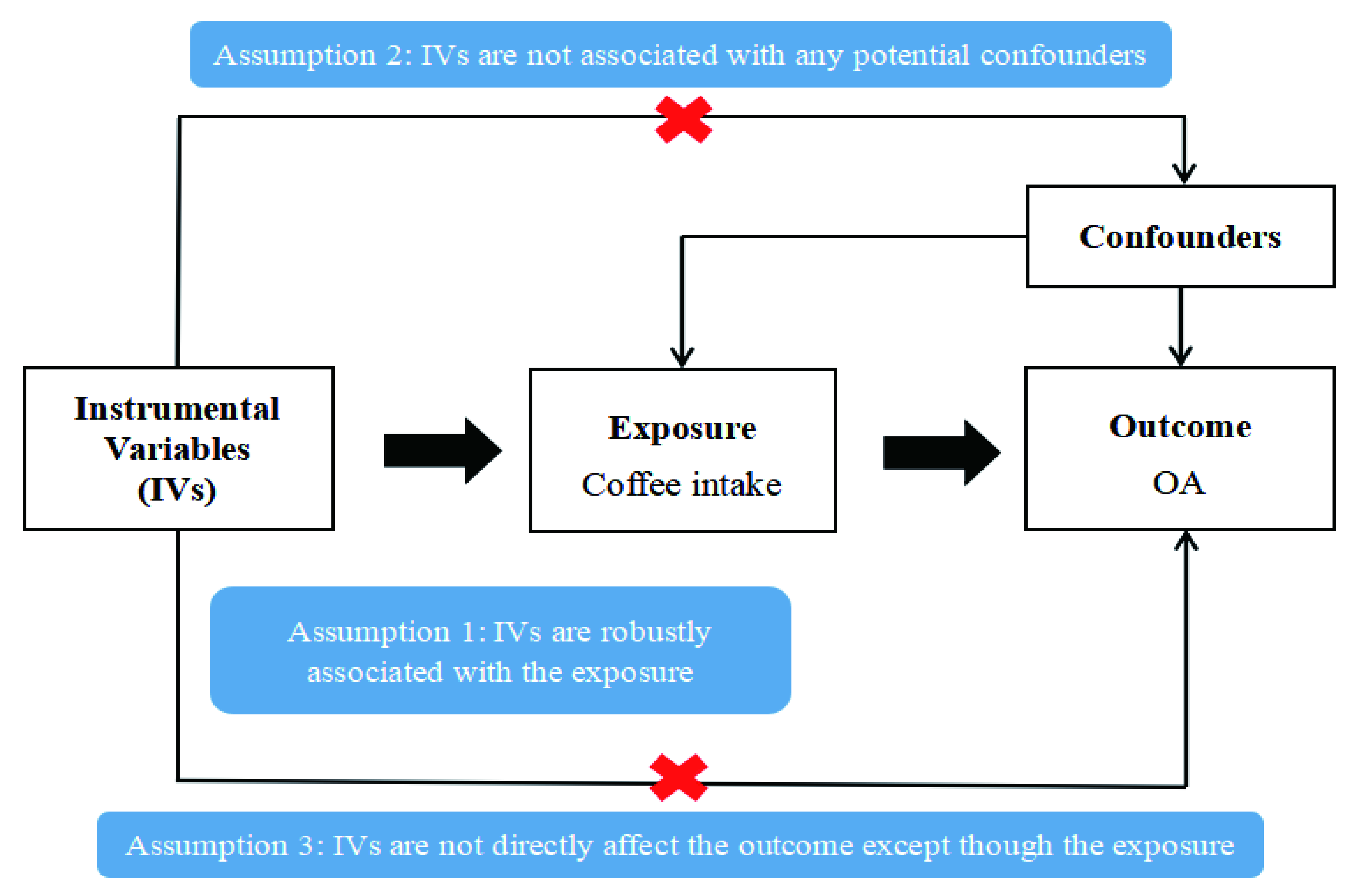

Supplement: Supplementary file 1 [file Data_Sheet_1.docx]
